# Mechanism of pod dehiscence in legumes: insights from phenotypic, anatomical, physiological and molecular studies

**DOI:** 10.7717/peerj.21676

**Published:** 2026-08-31

**Authors:** Bingjie Tu, Changkai Liu

**Affiliations:** 1 Key Laboratory of Heilongjiang Province for Cold-Regions Wetlands Ecology and Environment Research, Harbin University, Harbin, China; 2 State Key Laboratory of Black Soils Conservation and Utilization, Northeast Institute of Geography and Agroecology, Chinese Academy of Sciences, Harbin, China

**Keywords:** Dehiscence, Ventral suture, Gene, Hormones, Enzymes

## Abstract

Pod shattering describes the sudden splitting of dried mature legume pods to eject seeds *via* mechanical tension. Though vital for wild legume reproduction, it severely hinders crop harvesting and causes heavy yield losses, making it an unfavorable trait for cultivation. Pod shattering exists in legumes, grasses and brassicas, yet it is most prominent in legumes and a primary focus of crop domestication and breeding. This review analyzes the mechanism of pod shattering in legumes from the perspectives of morphology, anatomy of the pod suture zone, pod valve physiology, and molecular biology, with the aim of providing references for exploring practical strategies to alleviate pod shattering and for breeding cultivars resistant to pod shattering. The relationship between morphology and pod shattering in legumes varies substantially among different varieties, whereas the twisting phenomenon in common bean with longer pods deserves attention. Pod shattering in legumes mainly takes place at the pod ventral suture, modulated by soybean ventral fibrous cap cells, common bean valve margin cells and mesocarp lignification. Endogenous hormones in the pod suture stimulate hydrolases to degrade cell walls within the dehiscence zone and trigger shattering, while susceptible pods exhibit more pronounced fibrosis and lignification. Many legume pod-shattering genes such as *Pdh1, SHAT1-5, GmMYB26, PvPdh1* and* PvMYB26* have been widely studied. To clarify pod shattering mechanisms, we need to combine genetic improvement, plant structure adjustment and field management. Future work should integrate structural and physiological analyses of dehiscence zones to discover more shattering-related genes and develop practical field mitigation strategies, supporting the domestication and breeding of shatter-resistant legumes.

## Introduction

Wild plants engage in the indispensable process of seed dispersal to reproduce and sustain their offspring. Angiosperms, in particular, have evolved numerous novel seed dispersal mechanisms, enabling them to even utilize external vectors such as animals, wind, and water for seed dispersal (Funatsuki et al., 2014; Parker, Sassoum & Paul, 2021a). The evolution of resistance to pod shattering is one of the core domestication syndromes in legumes, equally important as the development of larger seeds, reduced seed dormancy, and decreased levels of toxins such as alkaloids in seeds (Hughes, Ringelberg & Bruneau, 2025). Legumes produce pods derived from a single carpel, and disperse seeds *via* a distinctive pod shattering mechanism. Upon maturity, the pod splits along both the ventral and dorsal sutures, releasing the seeds inside (Nathan & Muller-Landau, 2000; Funatsuki et al., 2014; Dong & Wang, 2015).

The economic value of legumes primarily derives from seed production, yet pod shattering can lead to devastating yield losses. However, yield loss due to pod shattering is affected by genotype, delayed harvesting, and environmental humidity (Agrawal, Patil & Salimath, 2002; Bhor, Chimote & Deshmukh, 2014). With the exception of a few species such as peanut (*Arachis hypogaea*) and carobs (*Ceratonia siliqua*), most legumes produce dehiscent fruits. Some grain legumes, including snow pea (a variety of *Pisum sativum*), edamame (*Glycine max*), mung bean (*Vigna radiata*), along with several forage legumes, are harvested while still green and fleshy (Sprent & James, 2008). However, seed losses caused by pod shattering during seed production have long been a persistent problem for breeders (Dong et al., 2017a; Murgia et al., 2017; Parker et al., 2020; Van der Merwe, Labuschagne & Smit, 2024; Zhang & Kyei-Boahen, 2007). Consequently, resistance to pod shattering has been preferentially selected as a crucial trait during the domestication of leguminous plants (Hughes, Ringelberg & Bruneau, 2025; Parker, Sassoum & Paul, 2021a).

Legume pods are derived from a single carpel consisting of two valves, with the central cavity enclosing the seeds. Parenchymatous tissues at the dorsal and ventral sutures connect the two valves, and it is at this region, known as the dehiscence zone (DZ), that the pod splits open at full maturity (Christiansen et al., 2002; Ferrándiz, 2002; Suzuki, Fujino & Funatsuki, 2009). Most research investigating pod shattering has centered on the dehiscence zone. In legumes, pod shattering is not induced by a single factor, its occurrence is influenced by phenotypic morphological characteristics, the anatomical structure of pod sutures, the chemical composition of pod walls, the genetic traits of cultivars, and environmental factors. The aim of this review is to systematically elucidate the functional roles of pod morphology, suture anatomy, pod wall components, suture-associated hormones and enzymes, as well as key genes, in pod shattering in legumes by using relevant literature.

## Survey Methodology

To ensure a comprehensive and objective overview of the field, we implemented a structured and iterative literature search strategy. The search process, encompassing database selection, search terms, and literature screening criteria, is detailed below.

**Search databases and scope:** The primary literature search was performed across three major academic databases to capture multidisciplinary research: Google Scholar, Web of Science, and Baidu Scholar. The search time frame was span-filtered from January 1, 1960, to February 28, 2026, to encompass both foundational classic studies and the latest research findings. To complement these databases and avoid missing relevant literature, cross-retrieval was supplemented by searching PubMed and the China National Knowledge Infrastructure (CNKI). Additionally, information was sourced from authoritative online resources, and the reference lists of key retrieved studies were manually checked to identify further relevant literature.

**Search terms and strategy:** Based on the core theme of this review, we utilized a combination of key terms including: “pod shattering”, “dehiscence”, “pod valve”, “ventral suture”, “anatomy”, “plant hormones”, “physiology”, “genes”, “legumes”, “twist”, and “dehiscence zone”. These terms were adapted using Boolean operators (AND/OR) to fit the specific retrieval rules of each database while maintaining consistency in core concepts.

**Literature selection guidelines:** Literature gathered from the search was carefully screened for inclusion in this review. Eligible studies primarily focused on peer-reviewed original research articles, comprehensive reviews, meta-analyses, and formally accepted preprints centered on pod shattering in legumes. Publications were restricted to those in English or with accessible comprehensive data, requiring a clear research design and complete methodological description. Conversely, we excluded non-peer-reviewed materials (such as editorials, conference abstracts, and book chapters), duplicate publications, retracted articles, or studies where core keywords appeared only incidentally without substantive discussion on legume dehiscence mechanisms.

## Pod Morphology

**Figure 1:**
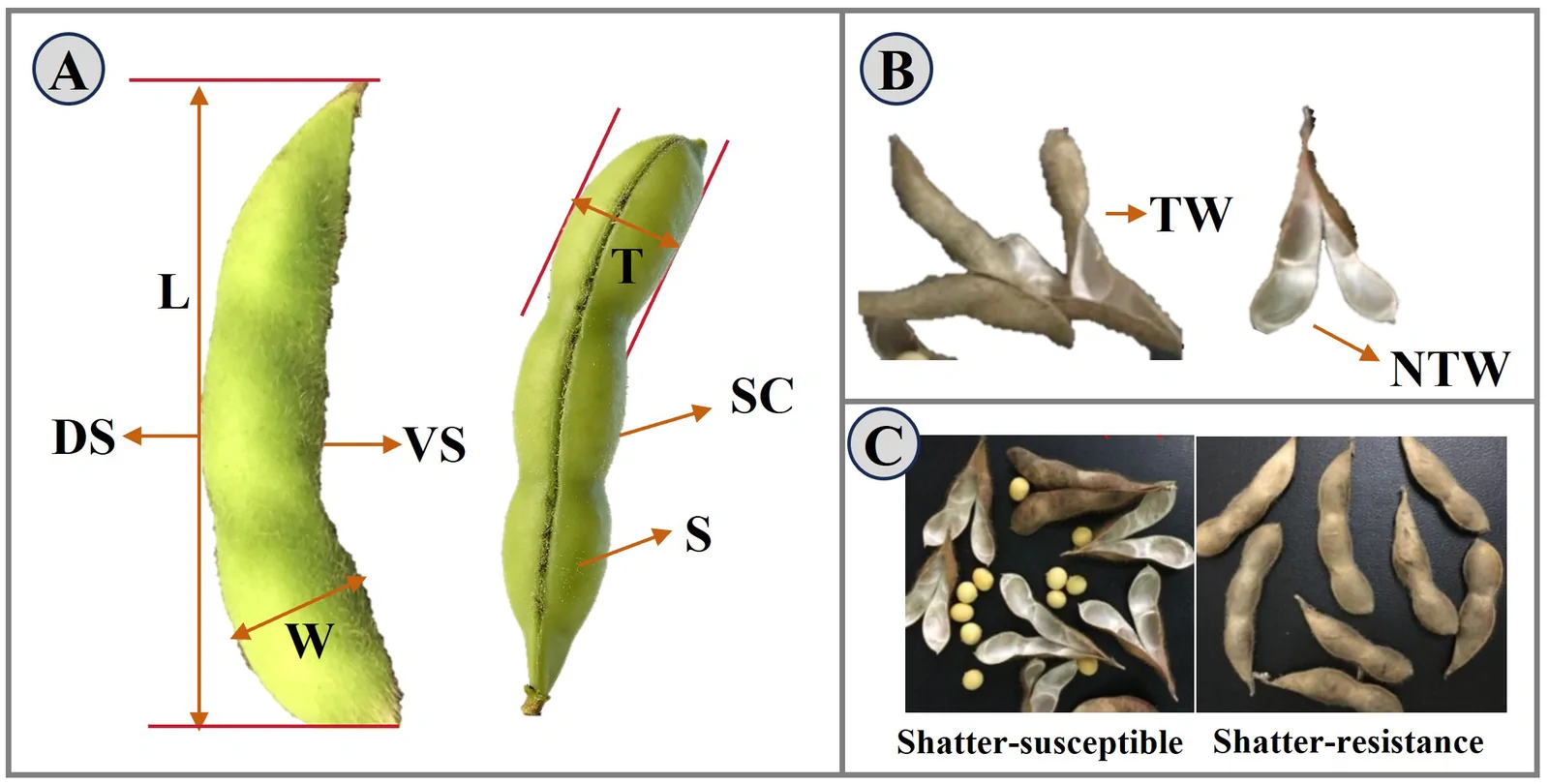
Soybean pod, lateral view (A). Pods of soybean with twisting and non-twisting phenotypes (B). Photographs of pod dehiscence in soybean (C). (A), (B) and (C) were photographed during our field experiments. DS, dorsal side; VS, ventral side; W, width; L, length; T, thickness; S, seeds; SC, single carpel; TW, twisting; NTW, non-twisting.

Leguminous pods, such as those of soybean, are all composed of carpels, sutures, and seeds (Fig. 1A). Legume pods vary greatly in size, ranging from small ones such as wild soybean (*Glycine soja*), vetch *(Vicia sativa* L.*)* and alfalfa *(Medicago sativa* L.*),* to medium-length pods including soybean (*Glycine max*), mung bean (*Vigna radiata* (L.) Wilczek) and pea (*Pisum sativum* L.), as well as long pods of common bean (*Phaseolus vulgaris* L.) and yardlong bean (*Vigna unguiculata* subsp. *sesquipedalis* (L.) Verdc.). Morphological traits of pods include pod length, width, thickness, curvature, and other related characteristics (Fig. 1A). Although pod morphology has long been considered an important determinant of pod shattering, current evidence across legume species remains inconsistent and species-dependent. In previous studies, the ratio of pod thickness to width in legumes has been considered a key trait during the pod dehiscence process (Caviness, 1969). No significant difference was observed in the ratio of pod thickness to width between shatter-susceptible and shatter-resistant vetch varieties (Dong et al., 2017b). Krisnawati & Adie (2017) reported that pod shattering was negatively correlated with the number of pods per plant, pod thickness, and the seed-to-pod weight ratio in soybean. A positive correlation between seed size and pod shattering tendency has also been observed in soybean (Tu et al., 2019). Pods with heavier pod walls and greater thickness exhibit stronger resistance to pod shattering, because the coiling degree of the pod wall is strongly influenced by its thickness (Fatima et al., 2023; Gioia et al., 2013; Takahashi et al., 2019). Some studies have also shown that pod shattering-resistant varieties exhibit a significantly lower ratio of pod thickness to width, whereas no significant differences in pod length, width, or thickness were detected in near-isogenic lines (NILs) (Tsuchiya, 1986; Suzuki, Fujino & Funatsuki, 2009). Researchers have even concluded that these pod morphological traits are not associated with pod shattering. In common bean, pod shattering comes with a “cost” characterized by small pods, low seed weight per pod, high pod weight, and a low seed-to-pod valve ratio (Murgia et al., 2017). In sharp contrast to this finding, the pod shattering syndrome in soybean is characterized by larger seeds, wider pods, and an open umbel-like plant architecture (Tu et al., 2025). The relationship between the morphological traits of legumes and pod shattering differs among varieties. No definitive conclusions have been reached regarding whether larger or smaller pods, as well as longer or shorter pods, are more prone to pod shattering.

Pod shattering typically results from tension generated in the pod wall (Estornell et al., 2013; Liljegren et al., 2004). Shattering occurs when the adhesive force between the two valves exceeds the tension of the pod wall. In Brassicaceae, the lignified layer at the margin of the pistil may generate considerable tension, promoting the detachment of petals from the stigma *via* a spring-like mechanism (Spence et al., 1996). Physical tension generated in the inner wall of legume pods causes morphological changes in the two valves. This is referred to as the chirality-creating mechanism, which transforms an initially flat pod valve into a helix in *Bauhinia* (Armon et al., 2011), and as twisting in cowpea (*Vigna unguiculata* L.) (Bijarniya et al., 2024) and common bean (Murgia et al., 2017). Figure 1B shows the morphology of twisting and non-twisting soybean pods. Armon et al. (2011) employed the mathematical framework of incompatible elasticity to model legume pods as thin strips with a flat intrinsic metric and saddle-shaped intrinsic curvature. Their work quantitatively predicted the observed configurations and linked the microstructure of pods to their macroscopic conformations. For phenotypic evaluation of pod shattering in legumes, in addition to describing the shattering percentage of samples, twisting was used as a screening index, and shattering was scored using weighted mean calculations (Bijarniya et al., 2024; Murgia et al., 2017). In fact, there is a gradual transition from non-twisting to twisting during pod shattering. To investigate the twisting phenotype associated with shattering in common bean, Indian researchers classified the types of shattering response in common beans into 10 graded scale: indehiscent, fissured, split, 20% twist, 40% twist, 60% twist, 80% twist, 100% twist, 80% abscised, and 100% abscised (Fatima et al., 2023). The helical coiling and rotational deformation of the two valves during pod dehiscence represents a highly intriguing research area. Compared with common bean and cowpea, soybean exhibits less frequent twisting due to its shorter pod length (Tu et al., 2025). Only a small proportion of soybean pods undergo twisting during dehiscence (Fig. 1B), and the typical shatter-susceptible phenotype observed in the field compare with shatter-resistance is shown in Fig. 1C. To achieve the separation of cells between the two valves in the dehiscence zone, the physical force that triggers cell detachment at the abscission layer (AL) is extremely important. Future studies should thoroughly analyze the relationship between the morphological traits of legume pods and the tension that triggers pod shattering, which will be of great significance for research on pod shattering.

## Anatomy of the Dehiscence Zone

The pods of leguminous plants are mostly flat and ribbon-shaped, tough in texture, with their appearance varying by variety, and there is no septum inside them. Two single carpel valves enclose the locule where seeds develop. The dorsal and ventral sutures connect the two valves, and the pod dehisces along these sutures upon maturity to release the seeds (Christiansen et al., 2002). In Brassicaceae plants, a narrow band of marginal cells exists along the two sutures of the silique, forming the DZ, which consists of approximately two cell layers. A non-lignified separation layer (SL) persists throughout silique development (Ferrándiz, 2002). This two-cell-wide structure of the DZ remains a non-lignified AL at maturity, and this parenchyma tissue facilitates silique dehiscence and the release of mature seeds (Meakin & Roberts, 1990). SL and DZ are also present in the sutures of leguminous plants, and have become key research areas for dissecting the genetic regulation of pod dehiscence and breeding shatter-resistant cultivars.

**Figure 2:**
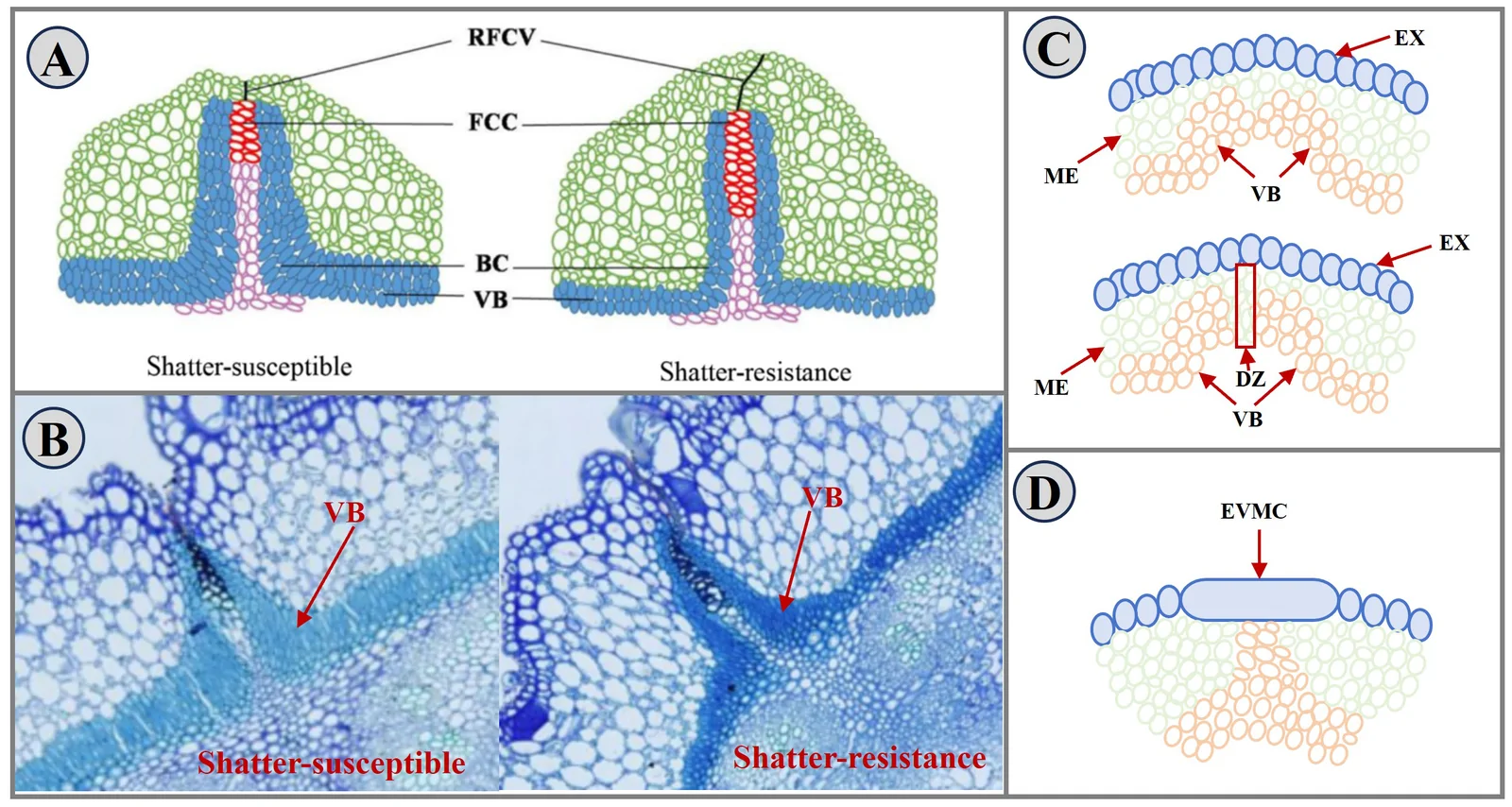
Fiber cap cells (FCC) in the ventral suture of soybean pod (A). The straight route from the top of fiber cap cells to the connecting point of the two valves (RFCV) structure in the soybean ventral suture (A). Morphology of the vascular bundles in the dehiscence zone of the ventral suture in shatter-resistant and shatter-susceptible soybeans (B). Comparing the presence and absence of the dehiscence zone (DZ) in the ventral suture of common vetch (C). External valve margin cells (EVMC) in the ventral suture of common vetch (D). (A) and (B) was cited from our previous work (Tu et al., 2019) ((C) was cited from Dong et al., 2017a) ((D) was cited from Dong et al., 2017b). (C) and (D) are drawn by the authors based on the literature. AL, abscission layer; VB, vascular bundle; BC, bundle cap; EX, exocarp cells; ME, mesocarp.

In the ventral suture of the soybean pod, there exists a fiber cap cell (FCC) region composed of several cell layers, which serves as an important cell band connecting the vascular bundles in the two valves (Fig. 2A). Compared with wild soybean, the significantly thickened FCC in the ventral suture of cultivated soybean pods represent a key breakthrough in soybean domestication, conferring resistance to pod shattering in cultivated soybean (Dong et al., 2014). Tu et al. (2019) confirmed this finding, observing that soybean cultivars with shorter FCC are more susceptible to pod shattering. In addition, shatter-susceptible soybean exhibits a shorter and straight route from the top of fiber cap cells to the connecting point of the two valves (RFCV), as well as larger vascular bundle area and larger cap-encasing region area (Fig. 2A). *Sh1* encodes a C2H2-type zinc finger transcription factor that represses the expression of *SHAT1-5*, reducing secondary wall thickness of FCC in the pod suture AL and thereby promoting pod shattering (Li et al., 2024b). The study found that the shattering-susceptible soybean has a larger vascular bundle area in the dehiscence zone of the ventral suture, and the arrangement of vascular bundle cells differs significantly from that of the shattering-resistant one (Fig. 2B) (Tu et al., 2019). However, it has also been reported that there are no significant structural differences in the dorsal suture dehiscence zone among soybean near-isogenic lines (NILs) (Suzuki, Fujino & Funatsuki, 2009).

The anatomical structure of the ventral suture DZ in common vetch is highly similar to that in soybean, except that the outermost layer consists of a layer of exocarp cells (EX), which is absent in soybean (Fig. 2C). In the pod ventral sutures of common vetch from shatter-susceptible accessions, the vascular bundles were separated into two parts by the DZ (Fig. 2C). In contrast, shatter-resistant accessions lacked a DZ, and their vascular bundles remained undivided (Dong et al., 2017a). In common vetch, FCC that adhere the two valves were not observed. Instead, a cell type named external valve margin cells (EVMC) was identified. EVMC are composed of two to four parallel-arranged cells, which are evenly distributed in the two fruit valves (Fig. 2D). Their outer cell walls are extremely thickened, whereas the inner cell walls remain unchanged (Dong et al., 2017b). Researchers speculated that FCC and EVMC are homologous structures. The excessively thickened cell walls of EVMC can greatly strengthen the adhesion between the two fruit valves, thereby preventing pod dehiscence.

In common bean, the ventral suture cells of wild-type genotypes exhibit extensive sclerification, whereas domesticated cultivars show the opposite pattern. Lignification of the inner layer of sclerenchymatic cells in the pod wall at the ventral suture is a key factor responsible for pod shattering in common bean (Murgia et al., 2017). Non-shattering in common bean is associated with the absence of a functional AL in the lateral sheath of the ventral suture. Loss of pod dehiscence in legumes arises from histological convergent evolution and results from selection at homologous genetic loci (Di Vittori et al., 2021). Pod dehiscence in cowpea and *Medicago ruthenica* is associated with a high degree of lignification, increased lignin content, and enhanced cell wall fiber deposition in the pod endocarp (Lo et al., 2021; Zhu et al., 2025). The presence of FCC cells and EX cells at the pod suture, as well as the existence of a DZ, are key determinants of pod shattering in legumes. Lignification in the pod endocarp also serves as an important factor underlying tension generation in the pod valves. Future studies on legume pod sutures should further identify novel structural features and integrate gene expression, physiology, and anatomical structure to continue elucidating the mechanisms underlying pod shattering in legumes.

## Pod Physiology and Shattering

Rapid dehydration of the pod valves generates tension, which acts as the physical driving force underlying pod shattering. Legume pods typically consist of two valves that tightly enclose the seeds, protecting them from damage during development (Bennett, Roberts & Wagstaff, 2011). Pods serve not only to protect the seeds, but green pods also act as photosynthetically active organs, supplying assimilates and nutrients to promote seed growth and regulating the partitioning of organic matter during seed filling (Thom et al., 2012; Sengupta et al., 2019). After the seeds are harvested, the remaining dry pods, due to their high fiber content, can be used as livestock feed, organic fertilizer for soil amendment, fuel, or raw materials for weaving and handicrafts. The relationships among the chemical composition, key enzymes, and plant hormones of the pod and pod shattering have long attracted research attention.

*Medicago ruthenica* with easy pod shattering exhibits a higher degree of lignification in the endocarp of the pod. The contents of lignin, cellulose, hemicellulose, and pectin are significantly higher than those in shatter-resistant cultivars, whereas the moisture content is significantly lower (Romkaew et al., 2008; Zhu et al., 2025). Tu et al. (2025) reported consistent results in soybean. The rapid increase in pod dehydration rate near harvest enhances the tension that triggers pod shattering. High fiber and lignin contents in pods are positively correlated with the percentage of pod shattering. In common bean, the contents of total fiber, lignin, hemicellulose and cellulose in shatter-susceptible pods are higher than those in shatter-resistant genotypes, and are positively correlated with carbon content (Murgia et al., 2017). In rapeseed (*Brassica napus* L.), hemicellulose content was a stable physiological indicator affecting pod shattering resistance (Kuai et al., 2016). In common vetch, the total lignin content of shatter-resistant accessions was significantly lower than that of shatter-susceptible accessions (Dong et al., 2025). At the pod level, a nearly consistent finding is that legumes prone to pod shattering have higher contents of cellulose, hemicellulose, and lignin in their pod. Pod fiber content may affect the tension that triggers pod shattering, while differences in the mechanical properties of pod lignification and changes in turgor pressure associated with fruit maturation are important factors influencing pod shattering (Liljegren et al., 2004; Tu et al., 2025).

**Figure 3:**
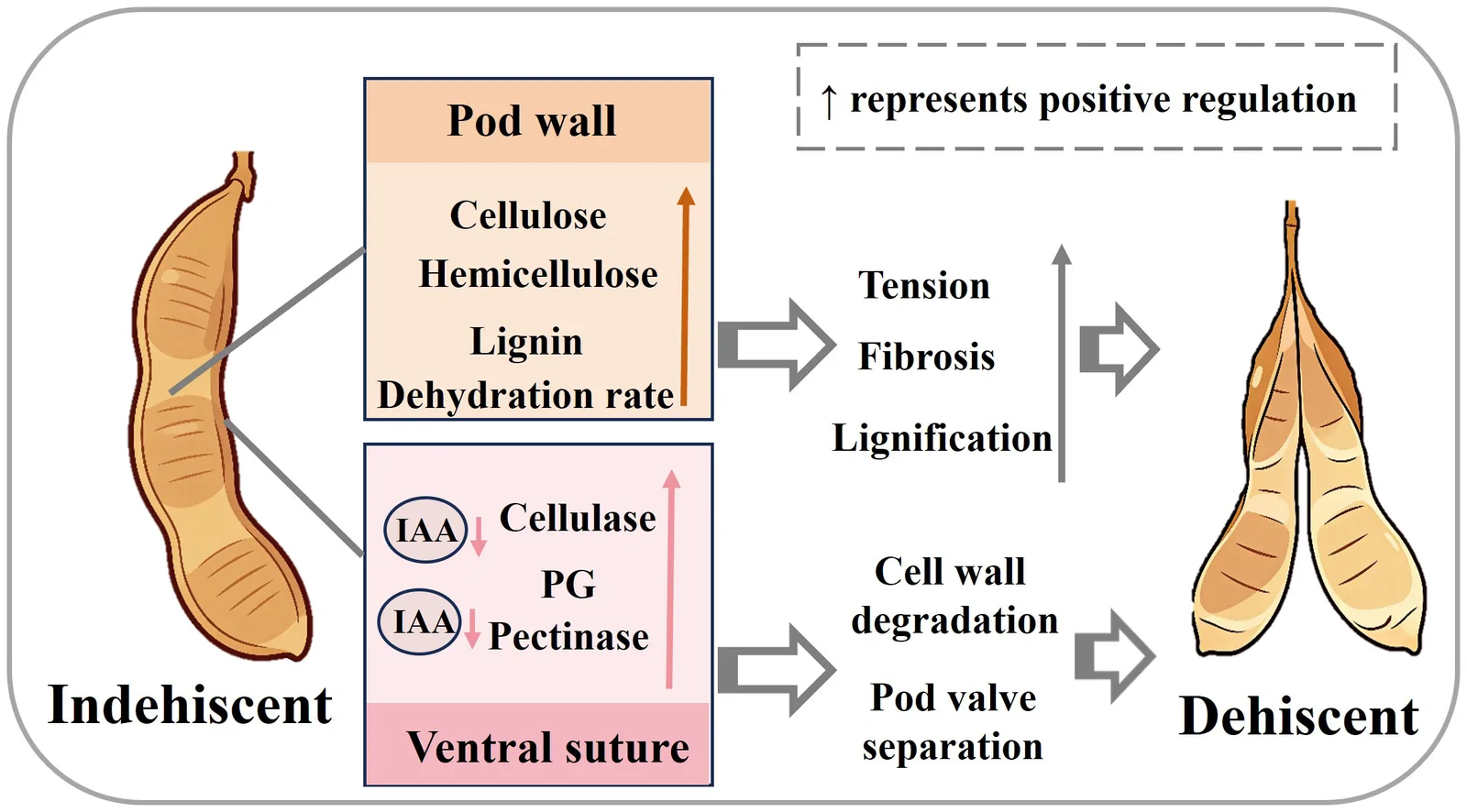
The physiological mechanisms underlying pod shattering at the pod and ventral suture levels. PG, polygalacturonase.

In the shatter-susceptible genotype of the legume *Medicago ruthenica*, increased activities of polygalacturonase (PG) and cellulase (CE) at the ventral suture of the pod are essential for the autolysis of AL cells (Guo et al., 2022). The degradation in the DZ of the pod is dependent on hydrolytic enzymes such as cellulase, pectinase, and PG (Petersen et al., 1996). In particular, endogenous 1,4-β-glucanase and polygalacturonase may disrupt the middle lamella of the separation layer, thereby reducing intercellular adhesion and consequently promoting pod shattering (Tsuchiya, 1986). The hydrolysis of galacturonan chains within pectin by pectinase and the degradation of cytoskeletal elements in the cell wall by cellulase collectively facilitate the breakdown of the middle lamella (Petersen et al., 1996; Brás et al., 2011; Hu et al., 2015). Phytohormones, particularly ethylene and indole-3-acetic acid (IAA), have been reported to be associated with pod dehiscence through their involvement in the regulation of cellulase enzyme activity (Tucker et al., 1988; Oeller et al., 1991; Maity et al., 2021). Furthermore, the role of IAA transport mediated by *Bna*. *WAG2* in reducing PG activity and enhancing pod shattering resistance through cell wall loosening was observed, which may reflect higher lignin accumulation and synthesis (Mahmood et al., 2023). Accumulating evidence has demonstrated that peroxidases play a crucial role in the polymerization of lignin precursors (Berthet et al., 2011). Cell wall peroxidases are capable of oxidizing the lignin precursor coniferyl alcohol, while higher concentrations of IAA exhibit an inhibitory effect (María et al., 1990). Hydrolysis of cell walls in the DZ depends on hydrolases, and the separation of AL cell walls is a combined result of cellulases, pectinases, and PG. The roles of plant hormones such as IAA, gibberellin (GA), and ethylene in the DZ, as well as whether exogenous application of plant hormones can affect the pod shattering phenotype, remain to be further investigated. The physiological mechanisms underlying pod shattering at the pod and ventral suture levels are summarized in Fig. 3. At the pod wall level, variations in the dehydration rate during desiccation of mature pod walls constitute a major source of the force driving pod dehiscence. Lignin deposition on cellulose and hemicellulose within pod walls promotes fibrous lignification of legume pods and elevates tensile stress for pod shattering. At the ventral suture level, elevated activities of cellulase, PG, and pectinase accelerate cell wall degradation in the dehiscence zone, which eventually leads to the separation of the two pod valves.

## Genes Related to Shattering

Research on pod dehiscence in soybean is more extensive than in any other leguminous plant, and the major genes exerting significant effects on this process have been identified (Parker, Sassoum & Paul, 2021a). A major pod dehiscence quantitative trait locus (QTL) has been finely mapped to chromosome Gm16 in the soybean genome (Bailey et al., 1997; Funatsuki et al., 2006; Yamada et al., 2009). Funatsuki et al. (2014) conducted map-based cloning to identify a major QTL governing pod dehiscence in soybean. Fine mapping and functional complementation demonstrated that this QTL encodes a dirigent-like protein, named *Pdh1*. The functional gene *Pdh1* was highly expressed in the lignin-rich inner sclerenchyma of pod walls, particularly during the early stage of lignin deposition. Subsequent studies on *Pdh1* and its association with pod shattering have been verified and further investigated in soybean (Miranda et al., 2019), chickpea (*Cicer arietinum* L.) (Aguilar-Benitez et al., 2020), cowpea (Marsh et al., 2023), and mung bean (Li et al., 2024a). Studies indicate that orthologous *Pdh1* genes originated specifically in warm-season legumes, and their loss-of-function (LoF) alleles underwent parallel selection during legume crop domestication (Yong et al., 2023). The domestication-related gene *SHAT1-5* on chromosome 16 promotes secondary wall biosynthesis and substantial thickening of FCC secondary walls *via* a 15-fold higher expression level than the wild-type allele, resulting from functional disruption of its upstream repressor (Dong et al., 2014). Another study demonstrated that *Sh1* encodes a C2H2-type zinc-finger transcription factor that promotes pod dehiscence by suppressing *SHAT1-5* transcription, thereby reducing secondary wall thickening in FCC of the pod suture abscission zone (Li et al., 2024b). Zhang & Singh (2020) identified a novel locus *NST1A* associated with soybean pod dehiscence *via* genome-wide association study (GWAS), alongside the previously reported *Pdh1* and *SHAT1-5* loci, and demonstrated that epistatic interaction between *Pdh1* and *NST1A* contributes critically to pod dehiscence resistance, while humidity shapes the distribution of indehiscent alleles. Studies have shown that quadruple mutations of *GmMYB26* confer enhanced resistance to pod shattering by tissue-specifically modulating the biosynthesis of lignin, cellulose, and hemicellulose (Takeshima et al., 2025).

Common bean is a highly versatile crop that was independently domesticated in Mesoamerica and the Andes. Multiple domestication events and extensive climatic adaptation have resulted in a diverse array of pod traits, making it exceptionally variable among legumes (Parker, Sassoum & Paul, 2021a). The *PvPdh1* locus located on chromosome Pv03 has been identified as a major regulator controlling pod shattering in common bean, with the *PvPdh1* allele functioning to reduce pod valve twisting (Parker et al., 2021b). Di Vittori et al. (2021) proposed *PvMYB26* is the most promising candidate gene underlying the major locus for pod indehiscence in common bean, supported by mapping and expression data that demonstrate its early and fine upregulation in dehiscent pods. More recently, studies have identified *PvMYB26* mutations in all three major gene pools of common bean, including an 8-kb deletion in Mesoamerican accessions; this mutant shows less than 1% of the wild-type expression level and a 44% decrease in lignin content. This research sheds light on the origins of agriculture in the Americas, suggesting that Ecuador and/or northern Peru likely represent the site of a third primary domestication of common bean, while west-central Mexico is the center of domestication and early agricultural emergence for Mesoamerican common beans (Celebioglu et al., 2026). Expression levels of *PlPdh1*, a homolog of *PvPdh1*, differ significantly between wild and domesticated lima bean (*Phaseolus lunatus* L.) (Garcia et al., 2021). QTLs associated with pod shattering have been extensively identified in other legumes, including cowpea, yardlong bean, and adzuki bean (*Vigna angularis*) (Kaga et al., 2008; Takahashi et al., 2020). *Pdh1*, *SHAT1-5*, *GmMYB26*, *PvPdh1*, *PvMYB26* and their homologs serve as the key genes governing pod shattering in legumes, and future efforts should focus on exploring novel genes and functionally characterizing known genes in depth.

Table 1 summarizes and presents key genes governing pod dehiscence in legumes along with their biological functions. Among these, the *pdh1* identified in soybean, which is associated with lignin deposition and pod twisting force during dehiscence, serves as the primary-effect gene regulating pod shattering in leguminous crops. The *pdh1* has been identified in common bean, cowpea, chickpea, mung bean, and lima bean. Recent studies over the past two years have revealed that *GmMYB26* from soybean and *PvMYB26* from common bean also function as vital genes regulating pod dehiscence in legumes. Subsequent investigations should clarify the upstream and downstream regulatory factors of the *pdh1*, *GmMYB26*, and *PvMYB26* pathways, and characterize functional differentiation among their homologous genes across various legume species.

**Table 1:** Genes related to pod shattering in legumes.

Legumes	Genes	Biological function and description	References
Soybean	*Pdh1*	Lignin deposition	Funatsuki et al. (2014); Miranda et al. (2019); Yong et al. (2023)
	*SHAT1-5*	Fiber cap cells	Dong et al. (2014)
	*NST1A*	Epistatic interaction	Zhang & Singh (2020)
	*Sh1*	fiber cap cells	Li et al. (2024b)
	*GmMYB26*	Fiber abundance	Takeshima et al. (2025)
Common bean	*PvPdh1*	Pod valve twisting	Parker et al. (2021b)
	*PvMYB26*	Functional abscission layer	Di Vittori et al. (2021); Celebioglu et al. (2026)
Cowpea	*Pdh1*	Lignin deposition	Marsh et al. (2023)
Chickpea	*Pdh1*	Lignin deposition	Aguilar-Benitez et al. (2020)
Mung bean	*Pdh1*	Lignin deposition	Li et al. (2024a)
Lima bean	*PlPdh1*	Homolog of *PvPdh1*	Garcia et al. (2021)

## Relationship Between Other Traits and Pod Shattering

Studies linking planting density to pod shattering in legumes are limited, whereas enhanced resistance to pod shattering with elevated plant density and row spacing has been documented in rapeseed, a member of the Brassicaceae family (Kuai et al., 2015). Foliar application of silicon to rapeseed plants elevates lignin accumulation, which in turn enhances resistance to pod shattering (Ahmad et al., 2022; Liu et al., 2024). While higher sugar levels have been detected in pods of shatter-susceptible vegetable soybean compared with shatter-resistant grain soybean cultivars (Tu et al., 2025), and dry matter accumulation and allocation are correlated with pod shattering (Agrawal, Patil & Salimath, 2002), the direct relationship between photosynthate translocation in pods, seeds and leaves of legumes and pod shattering still requires further investigation. Application of exogenous auxin significantly decreased pod shattering in shatter-susceptible cultivars to 23.6%, while concurrently downregulating peroxidase expression in *Medicago ruthenica* (Zhu et al., 2025). The umbellate, open plant architecture of soybean may be associated with shading and canopy closure, thereby influencing pod shattering (Tu et al., 2025). Environmental relative humidity is closely correlated with pod moisture content, thereby affecting the tension involved in pod shattering (Zhang et al., 2018). Future studies are still needed to explore field-applicable strategies for mitigating pod shattering, including fertilization management, planting density optimization, plant architecture improvement, and exogenous plant growth regulator application.

## Conclusions and Future Perspective

**Figure 4:**
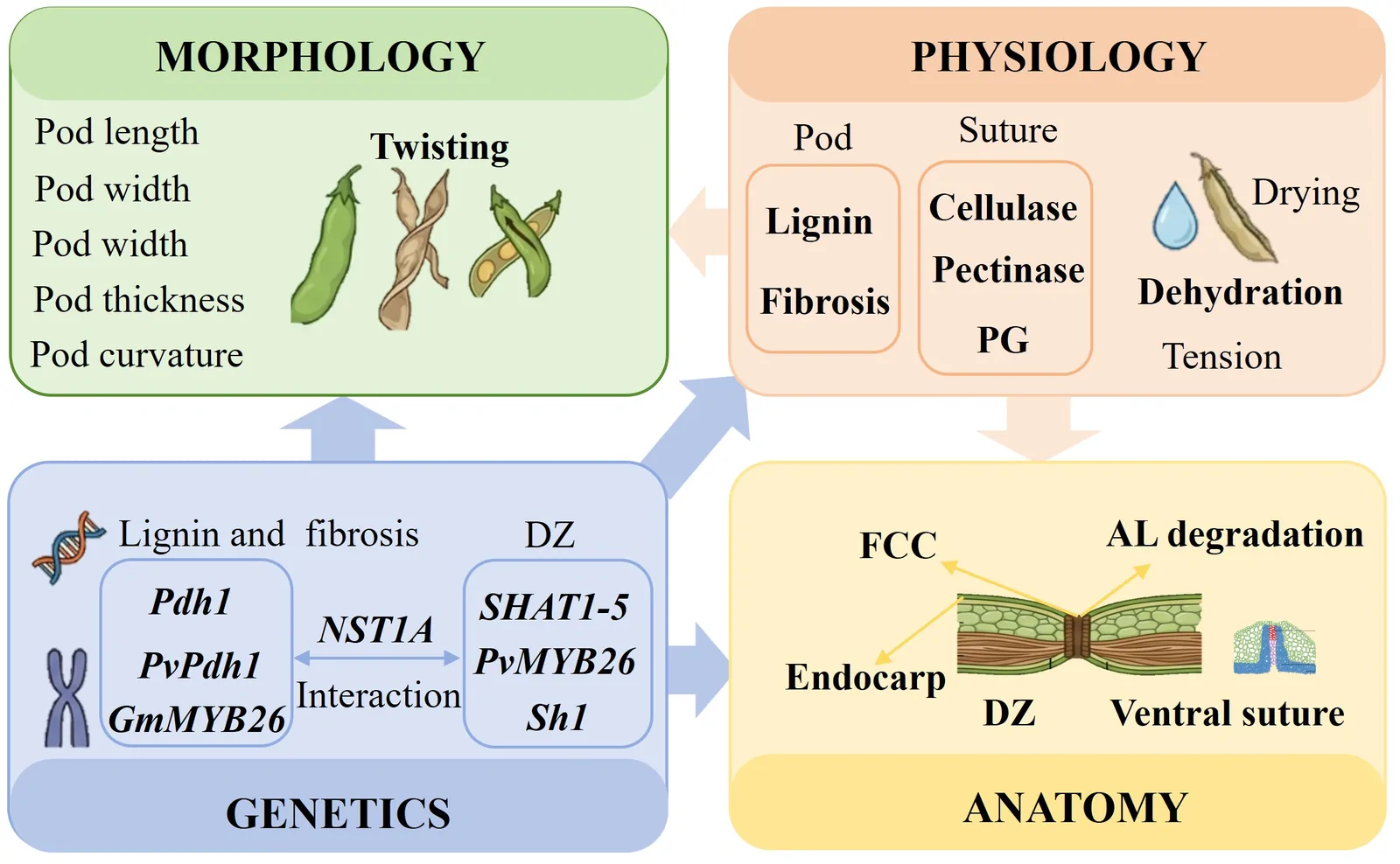
Integrated conceptual framework for legume pod shattering. PG, polygalacturonase; DZ, dehiscence zone; FCC, fiber cap cells; AL, abscission layer. This is a highly generalized conceptual framework diagram, while species-specific interspecific differences are elaborated in full detail within the main text.

Legume pod shattering is a complex process driven by the coordinated interaction of morphological, anatomical, physiological, and genetic factors. Diverse macro-morphological traits and micro-anatomical structures contribute to the accumulation of mechanical tension during rapid pod dehydration, whereas enzymatic degradation in the dehiscence zone (DZ) directly promotes cell-wall loosening and cell separation. These phenotypic, anatomical, and physiological changes are ultimately regulated by networks of key genes, highlighting the highly integrated nature of the pod-shattering mechanism. As summarized in Fig. 4, this review illustrates the relationships among these components, including gene-regulated phenotypic and anatomical characteristics, as well as the degradation and separation of abscission-layer cells driven by physiological changes in the pod suture. Building upon comprehensive overviews of legume pod shattering (Parker, Sassoum & Paul, 2021a; Liu et al., 2022; Liu et al., 2024), this review further emphasizes that although the fundamental biochemical mechanism of pod shattering is largely conserved, considerable morphological and genetic variation exists among different legume species. Therefore, it attempts to explore the general patterns and shared regulatory framework underlying pod shattering in legumes.

Future studies should focus on three major aspects. First, multi-layered regulatory pathways should be integrated to clarify the crosstalk between genetic networks and downstream morphological, anatomical, and physiological responses, thereby revealing how complex upstream signals jointly trigger pod dehiscence. Second, interspecific diversity should be further addressed through comparative genomics, transcriptomics, and functional verification across representative legume crops, with the aim of distinguishing conserved core mechanisms from species-specific regulatory innovations. Third, molecular breeding should be accelerated by mining elite haplotypes of core regulatory genes and developing efficient molecular markers, which will provide valuable genetic resources and theoretical guidance for breeding high-yield, shattering-resistant legume varieties suitable for mechanized harvesting

## Additional Information and Declarations

### Data Availability

The following information was supplied regarding data availability: This is a Literature Review.
